# CBD Alleviates Liver Injuries in Alcoholics With High-Fat High-Cholesterol Diet Through Regulating NLRP3 Inflammasome–Pyroptosis Pathway

**DOI:** 10.3389/fphar.2021.724747

**Published:** 2021-09-22

**Authors:** Xuye Jiang, Yingying Gu, Yuanling Huang, Yujia Zhou, Nengzhi Pang, Jing Luo, Zhaoyang Tang, Zhenfeng Zhang, Lili Yang

**Affiliations:** ^1^ Guangdong Provincial Key Laboratory of Food, Nutrition and Health, Department of Nutrition, School of Public Health, Sun Yat-sen University, Guangzhou, China; ^2^ Department of Nutrition, Binhaiwan Central Hospital of Dongguan, The Dongguan Affiliated Hospital of Medical College of Jinan University, Dongguan, China; ^3^ Guangdong Zhaotai Zinkernagel Biotech Co. Ltd, Foshan, China; ^4^ Key Laboratory of Nano-Immunoregulation Tumor Microenvironment, Department of Radiology, Translational Medicine Center and Guangdong Provincial Education Department, The Second Affiliated Hospital of Guangzhou Medical University, Guangzhou, China

**Keywords:** cannabidiol, alcohol liver disease, inflammation, NF-κB, nuclear factor kappa B, NLRP3 inflammasome, pyroptosis

## Abstract

Alcohol abuse and high-fat diet–induced liver diseases have been the most prevalent chronic liver diseases and the leading reasons for liver transplantation around the world. Cannabidiol (CBD) is a botanical component extracted from marijuana plants without psychoactive impact. In our previous reports, we found that CBD can prevent fatty liver induced by Lieber–DeCarli ethanol diet or non-alcoholic fatty liver disease (NAFLD) induced by high-fat high-cholesterol diet. The current work is a further study on whether CBD can alleviate liver injuries induced by ethanol plus high-fat high-cholesterol diet (EHFD), which is a model simulating heavy alcohol drinkers in a Western diet. A mice liver injury model induced by EHFD for 8 weeks was applied to explore the protective properties of CBD and the underlying mechanisms. We found that CBD prevented liver steatosis and oxidative stress induced by EHFD. CBD treatment inhibited macrophage recruitment and suppressed activation of NFκB–NLRP3–pyroptosis pathway in mice livers. The hepatoprotective property of CBD in the current model might be a result of inhibition of inflammation via alleviating activation of the hepatic NFκB–NLRP3 inflammasome–pyroptosis pathway by CBD.

## Introduction

Prolonged heavy consumption of alcohol-induced liver injuries accounts for the increasing morbidity and mortality rates worldwide (Xu et al., 2017). These liver injuries are characterized by a spectrum of progressing liver disorders, including simple fatty liver, alcoholic hepatitis, cirrhosis, and hepatocellular carcinoma (Seitz et al., 2018). Individuals abusing alcohol are usually accompanied with a superfluous calorie intake which will raise their predisposition of developing a metabolic disorder such as obesity, metabolic syndrome, and type 2 diabetes mellitus (T2DM) (Boyle et al., 2018). It has been recognized for many years that chronic heavy alcohol drinking can have synergistic or exacerbating effects on obesity and metabolic syndrome, which can further promote the formation of fatty Rinella (2015a) liver and aggravate the progression of ALD (Boyle et al., 2018) (Rinella, 2015b). Haein et al. proved that significant hepatic damage can be caused by alcohol administration for four weeks with increased hepatic lipid droplets and inflammatory cytokine levels and can be aggravated with the combination of high-fat diet (Kim et al., 2016). In the present study, we applied chronic administration of alcohol with a high-fat high-cholesterol diet (EHFD) to induce hepatic injury in mice.

Inflammation plays an important role in the formation and progress of liver damage induced either by alcohol or high-fat diet. Pyroptosis is an inflammatory process of caspase-1–dependent programmed cell death. In pyroptosis, the inflammasome and some other signals are activators of caspases. In general, pyroptosis starts with inflammasome formation recognizing various exogenous and endogenous signals, including LPS and ATP, and caspase-1 is subsequently activated. The activation of caspase-1 by the NOD-like receptor pyrin domain containing 3 (NLRP3) inflammasome leads to the cleavage of pro-inflammatory cytokine interleukin-1 beta (IL-1β) and pro-interleukin 18 (IL-18) and the production of mature IL-1β and IL-18. In addition, activated caspase-1 cleaves the pyroptotic substrate gasdermin D (GSDMD) and forms the membrane pores and induces pyroptosis, allowing the release of IL-1β and IL-18.

In canonical pyroptosis, inflammasome formation is an initiation step for pyroptosis activation. The inflammasome is an inflammatory signal platform, which can react with exogenous and endogenous signals and initiate a series of inflammatory responses (Fabio et al., 2002). The inflammasome is a cytoplasmic complex, mainly composed of three different functional proteins: sensor proteins, including a nucleotide-binding oligomerization domain (NOD)–like receptors (NLRs) family members; the adapter protein comprised apoptosis-associated speck-like protein containing a casp recruitment domain (ASC)— the effective protein pro-caspase-1 (Fabio et al., 2002; Benetti et al., 2013). Among the inflammasomes, studies have highlighted the importance of the NLRP3 inflammasome in many inflammatory disorders (Benetti et al., 2013), such as ALD, obesity (Mastrocola et al., 2018), type 2 diabetes (Luo et al., 2017), non-alcoholic fatty liver disease (NAFLD) (Wan et al., 2016; Cabrera et al., 2017), and metabolic syndrome (Mastrocola et al., 2018). The NLRP3 inflammasome can be activated by endogenous or exogenous factors, including pathogen-associated molecular patterns (PAMPs), damage-associated molecular patterns (DAMPs), pore-forming toxins, and environmental irritants (Fabio et al., 2002). Nuclear factor κB (NF-κB) has been demonstrated as one of key factors activating the NLRP3 inflammasome. Upon activation of the NLRP3 inflammasome, caspase-1 is activated. Activated caspase-1 would cleave pro-IL-1β into mature IL-1β, resulting in release of IL-1β, inflammation, and cell and tissue damage (Fabio et al., 2002). In addition, activated caspase-1 cleaves GSDMD and forms membrane pores, which is a key step for pyroptosis, allowing the release of IL-1β and IL-18.

Excessive activation of inflammatory caspases is implicated in the pathogenesis of alcoholic liver disease and NASH, and pyroptosis is the dominant response following this activation (Jorgensen and Miao, 2015). Heo et al. proved that pyroptosis occurs in hepatocytes with alcohol exposure as NLRP3, ASC, and caspase-1 were all upregulated with ethanol (Heo et al., 2019). Numerous studies have shown that pyroptosis is an inflammatory link between simple steatosis and NASH, since no NLRP3 activation was observed in simple steatosis without inflammation, while NLRP3 activation in NASH has been shown in both human and animal models (Csak et al., 2011; Beier and Banales, 2018). Moreover, researchers had proved that the pyroptosis-inducing fragment GSDMD expression was higher in NASH and that IL-1β released following pyroptosis is the driver of liver inflammation and fibrosis (Tilg and Moschen, 2011; Shi et al., 2015).

Cannabidiol (CBD), a non-psychomimetic compound extracted from *Cannabis sativa*, exerts a broad range of pharmacological properties, including cardiac and neural protection, antioxidant and anti-inflammatory capabilities, and anti-epileptic potential (Yang et al., 2014; Wang et al., 2017; Huang et al., 2019). Although CBD can cross the blood–brain barrier, it does not cause psychoactive effects that lack abuse potential. CBD has been shown to reduce the inflammation in cultured human sebocytes and human skin organ culture through coupling to A2A adenosine receptor–dependent upregulation of tribbles homolog 3 (TRIB3) and inhibition of NF-κB signaling (Oláh et al., 2014). Moreover, the intervention of CBD delayed the onset of type 1 diabetes in non-obese diabetic mice and alleviated pancreas inflammation (Lehmann et al., 2016). It has also been shown that the administration of CBD targeted NF-κB and NLRP3 inflammasome pathways in macrophages, which could be a novel treatment for NASH (Mridha et al., 2017). In addition, CBD treatment suppressed hepatic lipid accumulation and attenuated oxidative stress and inflammatory response induced by excess energy diet Huang et al. (2019) or alcohol intake (Wang et al., 2017). Recent studies have shown that CBD was able to reduce accumulation of intracellular lipid levels (Silvestri et al., 2015) and cell death (Mukhopadhyay et al., 2011) in dose- and time-dependent manners. It is still elusive whether CBD can protect liver injury induced by EHFD, and the related mechanisms are unclear. Here, we aim to explore the potentiality of CBD for mitigating liver inflammation induced by EHFD, as well as the mechanism therein, focusing on the NLRP3 inflammasome–pyroptosis pathway in macrophages.

## Materials and Methods

### Reagents

Cannabidiol (CBD) was from Tocris Bioscience (Ellisville, MO, United States, 1570/10), with purity ≥98% (HPLC). TRIzol was supplied by Invitrogen (Carlsbad, CA, United States). The PrimeScript RT Reagent Kit with a gDNA eraser and SYBR Premix Ex TaqII kit were from TaKaRa (Tokyo, Japan). Lysis buffer for Western blot and a BCA protein assay kit were purchased from Beyotime Biotechnology Company (Shanghai, China). Anti-pIκBα (9246s), anti-IκBα (4812s), anti-pNF-κB p65 (3033s), anti-NF-κB p65 (8242s), anti-ASC (#67824), and anti-IL-1β (#12242) were from Cell Signaling Technology. Anti-NLRP3 (ab214185) and anti-tubulin (ab6160) were obtained from Abcam. Anti–pro-caspase-1 (22915-1-AP) and anti-GSDMD (20770-1-AP) were obtained from Proteintech Group Inc. (United States). Anti-GAPDH (sc25778), anti–caspase-1 p20 (sc25778), and all secondary antibodies were purchased from Santa Cruz Biotechnology (Santa Cruz, CA, United States).

### Animal Experimental Protocol

**FIGURE 1:**
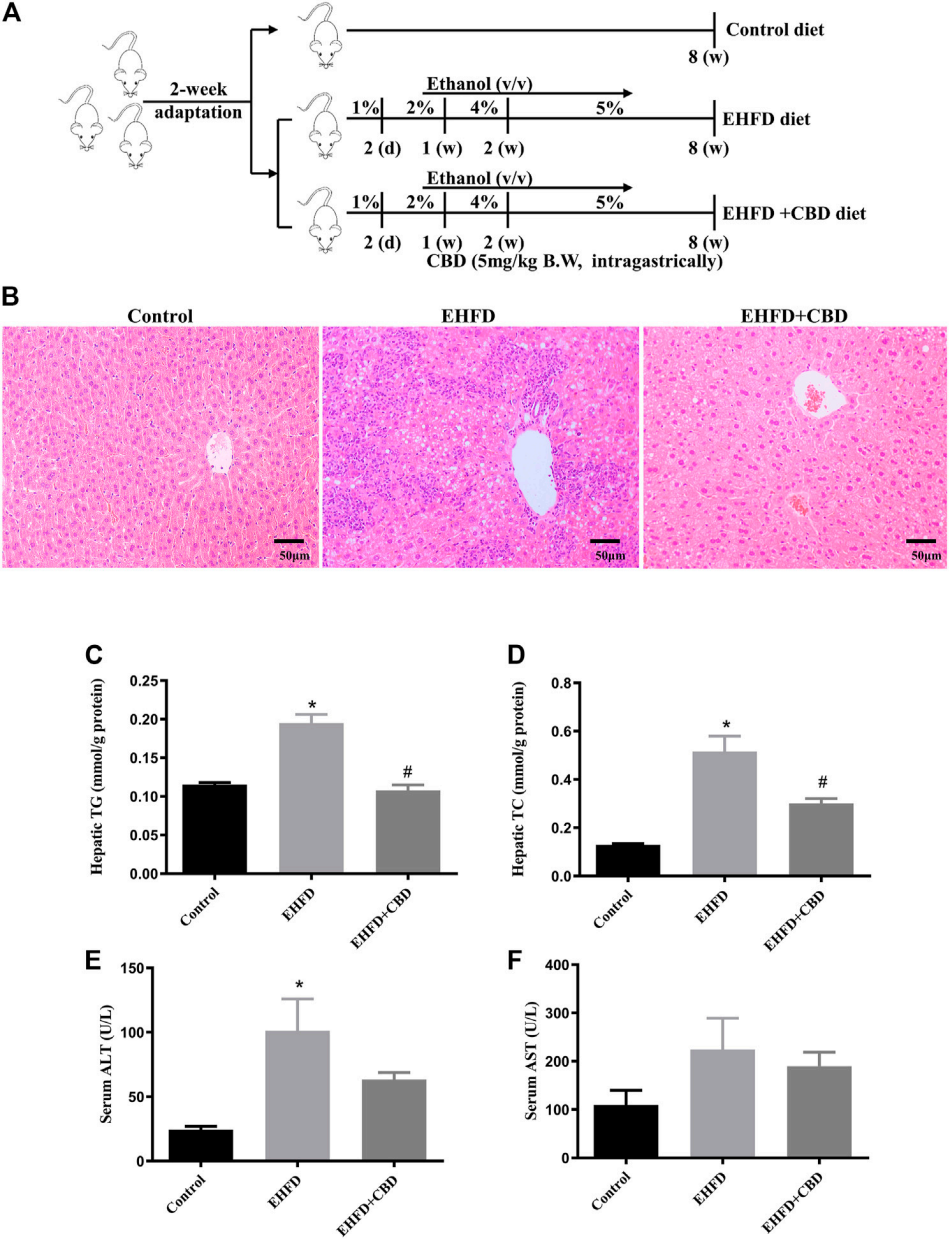
CBD treatment ameliorated steatohepatitis induced by EHFD diet. Mice were fed ethanol plus high-fat high cholesterol diet for 8 weeks with or without CBD treatment. Control mice were fed a standard chow diet. The animal experiment protocol **(A)**. Representative H&E staining of liver sections (magnification ×200) **(B)**. Levels of hepatic TG **(C)** and TC **(D)**. Levels of serum ALT **(E)** and AST **(F)**. Values represent mean ± SEM (*n* = 6–8/group). *p < 0.05 vs the control group; #*p* < 0.05 vs the EHFD group.

Six-week-old male C57B/6J mice were supplied by the Sun Yat-sen University Animal Center (Guangzhou, China). Animal experiments were approved by the Animal Experimentation Ethics Committee of Sun Yat-sen University, complied with the ARRIVE guidelines and the National Institutes of Health Guide for the Care and Use of Laboratory Animals. All mice were kept at a specific pathogen‐free environment (temperature 20–22°C, humidity 60%) with 12-hour light/dark cycle and free access to food and water. After two weeks of acclimatization, the mice were randomly assigned to the following three groups: 1) the control group, received a standard chow diet (5% fat w/w); 2) the EHFD group, received a high-fat high-cholesterol diet (containing 17% fat and supplemented with 1.25% cholesterol and 0.5% cholate) which was obtained from Trophic Animal Feed High-tech Co. Ltd. (China) Gäbele et al. (2011), with *ad libitum* access to alcohol in drinking water with increasing concentrations of alcohol (1% v/v of alcohol for the first 2 days, 2% from day 3 to day 7, 4% for the second week, and 5% for another 6 weeks); and 3) the EHFD + CBD group, on the basis of EHFD group, mice were gavaged with 5 mg/kg BW CBD solution per day for 8 weeks. The control and EHFD mice were given saline at equivalent volumes. The animal experiment protocol is shown in Figure 1A. The mice were euthanized at the age of 16 weeks after they were fasted for 16 h, and the serum and liver tissue were harvested for subsequent measurement.

### Biochemical Analysis

The serum was collected after 3000×g centrifugation at 4°C for 15 min. Levels of serum aspartate aminotransferase (AST), serum alanine aminotransferase (ALT), hepatic triglyceride (TG), and hepatic total cholesterol (TC) were determined by corresponding kits (Jiancheng Biotech, Nanjing, China) according to the manufacturer protocol. Hepatic malondialdehyde (MDA) levels, glutathione/glutathione disulfide (GSH/GSSG) levels, and activity of superoxide dismutase (SOD) were determined by using ELISA kits (Beyotime, Shanghai, China). The values were normalized by protein content.

### Histology and Immunohistochemical Analysis

Liver tissues were fixed in 4% paraformaldehyde and embedded in paraffin. Cross sections (5 µm) of the tissue were cut and then mounted on glass for histology and immunohistochemical analysis. Hematoxylin and eosin (H&E) staining was performed to assess liver tissue morphology. Immunohistochemical staining was described as before (Lv et al., 2019). In brief, the liver tissues were embedded in paraffin and sliced into 5-μm sections. After deparaffinization and rehydration, the sections were incubated in sodium citrate buffer. Then 1% H_2_O_2_ was used to suppress the endogenous peroxidase activity. Thereafter, the sections were incubated with an anti-CD68 antibody at 4°C overnight. The sections were then incubated with a secondary antibody—anti-rabbit IgG (Santa Cruz, CA, United States) for 1 h at room temperature. Finally, immunospecific reactivity was visualized by diaminobenzidine (DAB; ZSGB-BIO, Beijing, China) substrate and counterstained with hematoxylin (Jiancheng Biotech, Nanjing, China). Images were acquired with a microscope (Leica, Solms, German).

### Western Blot Analysis

The proteins extracted from the liver tissues were analyzed by Western blot. The liver tissues were homogenized using RIPA (radioimmunoprecipitation assay) lysis buffer supplemented with protease and phosphatase inhibitors. The proteins were separated by 8–12% SDS-PAGE, followed by transferring to a 0.22/0.45-mm polyvinylidene difluoride (PVDF) membrane (Millipore, Billerica, MA, United States). The membranes were pre-blocked with 5% (w/v) fat-free milk in phosphate-buffered saline containing 0.05% (v/v) Tween-20 (TBST). The membrane was incubated at 4°C overnight with specific primary antibodies and subsequently incubated with HRP-conjugated secondary antibodies at room temperature for 1 h. The ECL Reagent Kit (Thermo) was used to determine signals. Primary antibodies included anti-pIκBα, anti-IκBα, anti-NF-κB p65, anti-pNF-κB p65, anti-NLRP3, anti-ASC, anti–pro-caspase-1, anti–caspase-1 p20, anti-GSDMD, anti-IL-1β, anti-GAPDH, and anti-tubulin. The band intensities were analyzed by ImageJ software.

### Quantitative Real-Time Polymerase Chain Reaction

Total RNAs were extracted from livers with TRIzol reagent and reverse-transcribed into cDNA with a PrimeScript RT Reagent Kit. Gene expression was determined by quantitative real-time PCR with an SYBR Premix Ex Taq II kit and signaling detected by a Vii7 system (ABI, Carlsbad, CA, United States). Expression of each target gene was normalized to the internal standard (β-actin) by using the 2^−ΔΔCt^ comparative method. Primer sequences are listed in Table 1.

**TABLE 1:** Primer sequences for quantitative real-time polymerase chain reaction.

IL-1β	Forward	5’-TCA​CAG​CAG​CAC​ATC​AAC​AA-3’
	Reverse	5’-TGT​CCT​CAT​CCT​GGA​AGG​T-3’
MCP-1	Forward	5’-TGC​CCT​AAG​GTC​TTC​AGC​AC-3
	Reverse	5’-AAG​GCA​TCA​CAG​TCC​GAG​TC-3’
TNF-α	Forward	5’-GCT​CTT​CTG​TCT​ACT​GAA​CTT​CGG-3’
	Reverse	5’-ATG​ATC​TGA​GTG​TGA​GGG​TCT​GG-3’
F4/80	Forward	5’-TGA​CTC​ACC​TTG​TGG​TCC​TAA-3’
	Reverse	5’-CTT​CCC​AGA​ATC​CAG​TCT​TTC​C-3’
β-actin	Forward	5’-CGT​GAA​AAG​ATG​ACC​CAG​ATC​A-3’
	Reverse	5’-CAG​CCT​GGA​TGG​CTA​CGT​ACA -3’

### Statistical Analysis

All statistical analyses were performed with SPSS 20.0, and a *p* value < 0.05 was considered statistically significant. Data were expressed as means ± SE analyzed by Student’s *t*-test (for two groups) or one-way ANOVA (for more than two groups), followed by the Bonferroni test.

## Result

### CBD Treatment Ameliorated Steatohepatitis Induced by EHFD Diet

As shown in Figure 1B, EHFD diet resulted in marked hepatocellular lipid accumulation with obvious inflammatory cell infiltration in mice liver as compared with those in control, while CBD treatment markedly decreased lipid droplets and inflammatory lesions induced by EHFD in H&E-stained liver sections. Correspondingly, we found that the levels of TG and TC in the liver increased in the EHFD group (Figures 1C, D), with significantly increased serum ALT and AST levels (Figures 1E, F), indicating the development of steatohepatitis and the damage of liver cells in EHFD treatment. CBD treatment significantly inhibited hepatic steatohepatitis and liver injury induced by EHFD diet.

### CBD Treatment Alleviated Oxidative Stress Induced by EHFD Diet

**FIGURE 2:**
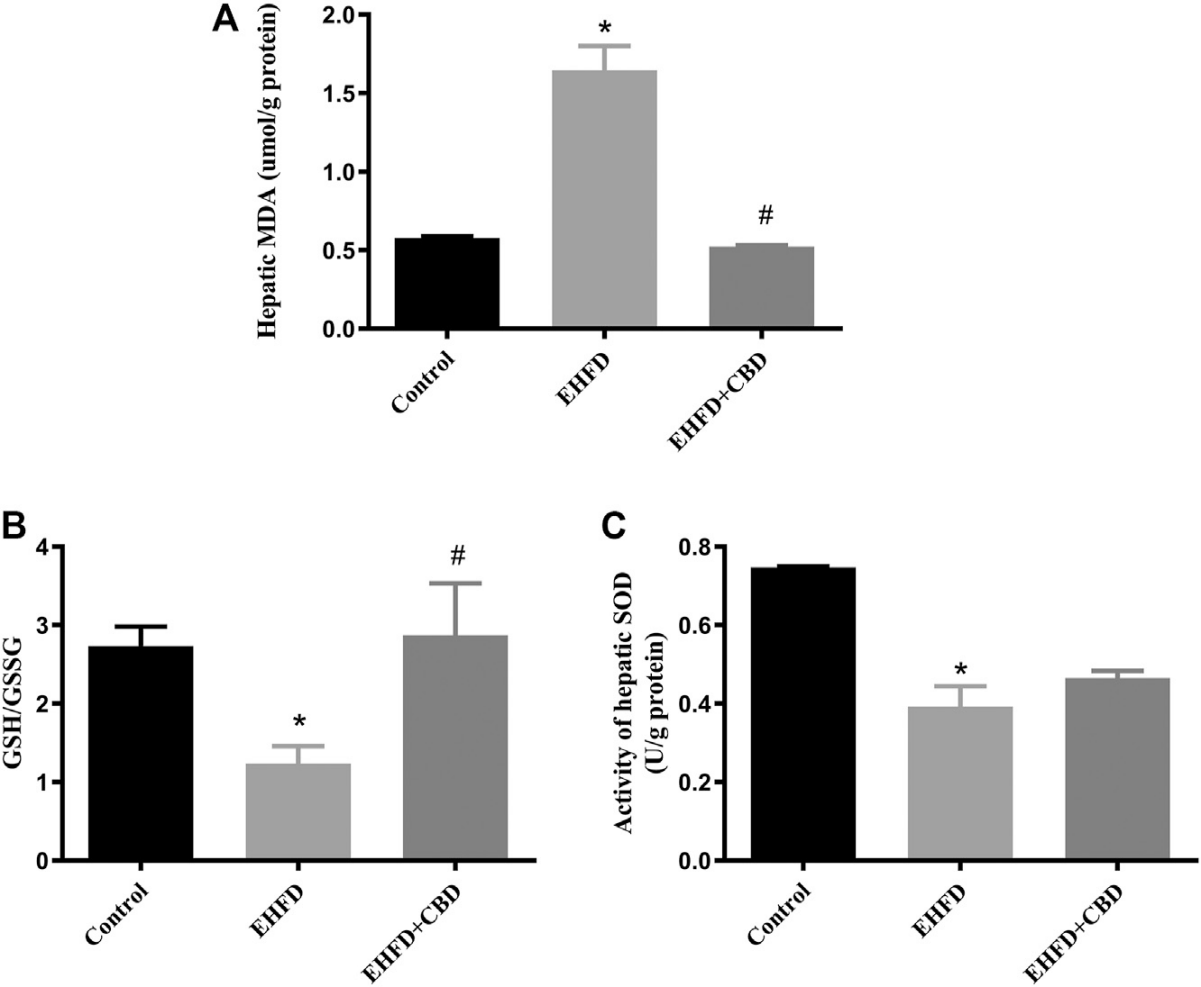
CBD treatment alleviated oxidative stress induced by EHFD diet. Mice were fed ethanol plus high-fat high cholesterol diet for 8 weeks with or without CBD treatment. Control mice were fed a standard chow diet. Levels of hepatic MDA **(A)**. The ratio of hepatic GSH/GSSG **(B)**. Activity of hepatic SOD **(C)**. Values represent mean ± SEM (*n* = 6–8/group). *p < 0.05 vs the control group; #*p* < 0.05 vs the EHFD group.

Excess of oxidative stress contributes to cellular injury and the development of non-alcoholic and alcoholic liver disease. To evaluate the effect of CBD on oxidative stress, we measured the activity of enzymes that maintain redox homeostasis and the level of lipid peroxidation product in mice liver. Figures 2A, B show that the mice in the EHFD group displayed significantly increased contents of MDA and a decreased ratio of GSH/GSSG, whereas these alterations were inhibited in the presence of CBD. Moreover, EHFD decreased hepatic activity of SOD, which was slightly increased by CBD treatment, with no statistical significance compared to the EHFD group (Figure 2C).

### Inflammation Induced by EHFD Diet Was Ameliorated by CBD Treatment

**FIGURE 3:**
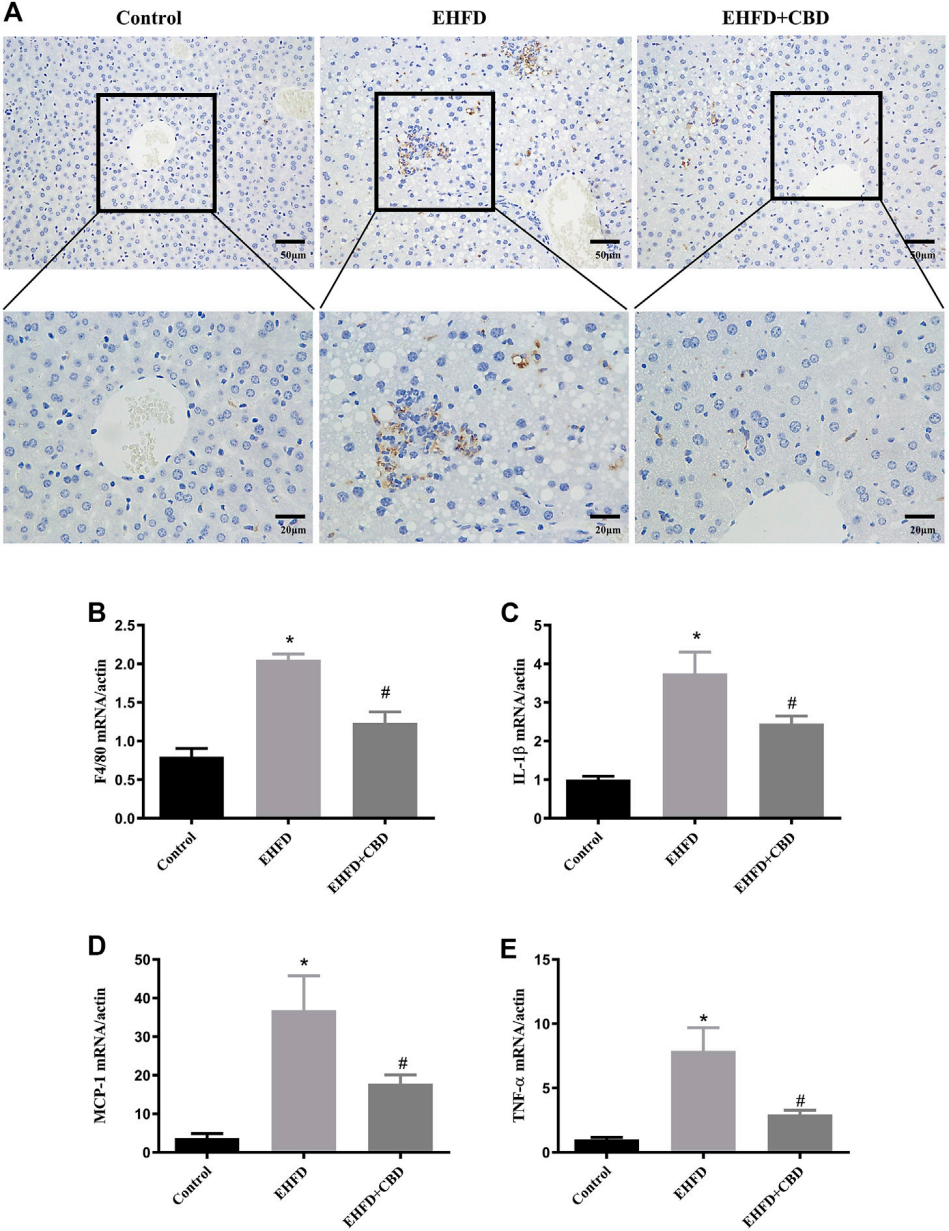
Inflammation induced by EHFD diet was ameliorated by CBD treatment. Mice were fed ethanol plus high-fat high cholesterol diet for 8 weeks with or without CBD treatment. Control mice were fed standard chow diet. Immunohistochemistry staining was performed to determine CD68^+^ macrophages (magnification ×200 and ×400) **(A)**. The mRNA expressions of F4/80 **(B)**, IL-1β **(C)**, MCP-1 **(D)**, and TNF-α **(E)** were analyzed by qPCR. Values represent mean ± SEM (*n* = 6–8/group). *p < 0.05 vs the control group; #*p* < 0.05 vs the EHFD group.

The H&E-stained liver sections revealed that CBD treatment decreased hepatic inflammation in EHFD mice. To further verify the anti-inflammatory effect of CBD, we assessed the expression of CD68 (macrophage marker) in mice liver. Immunohistochemistry staining of liver sections indicated that number of CD68-positive cells was increased in the EHFD group, suggesting an increased inflammatory cell infiltration. The expression of CD68 was blunted in the CBD group (Figure 3A). Consistently, gene expressions of the inflammatory markers such as F4/80, IL-1β, monocyte chemoattractant protein-1 (MCP-1), and tumor necrosis factor alpha (TNF-α) were increased in the EHFD group, while these gene expressions were significantly reduced with CBD treatment (Figures 3B–E). These results indicated that administration of CBD alleviated the inflammatory response induced by EHFD.

### CBD Treatment Inhibited the Activation of NF-κB Pathway Induced by EHFD Treatment

**FIGURE 4:**
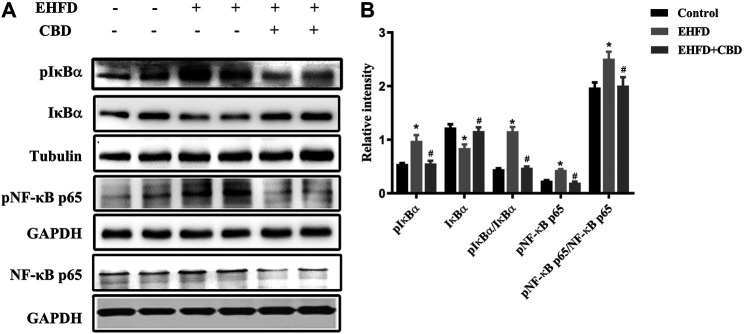
CBD treatment inhibited NF-κB pathway activation induced by the EHFD diet. Mice were fed ethanol plus high-fat high-cholesterol diet for 8 weeks with or without CBD treatment. Control mice were fed a standard chow diet. Western blot analyses of pIκBα, IκBα, pNF-κB p65, and NF-κB p65 **(A)**. Quantification of expressions of IκBα, pIκBα, and pNF-κB p65 **(B)**. Values represent mean ± SEM (n = 6–8/group). *p < 0.05 vs the control group; #*p* < 0.05 vs the EHFD group.

NF-κB is a key transcription factor that controls the expression of pro-inflammatory genes. To determine the possible involvement of the NF-κB pathway in the anti-inflammatory effect of CBD, we examined IκBα and NF-κB in the liver samples by Western blot. As shown in Figure 4, IkBa was decreased due to its phosphorylation in the EHFD group, EHFD treatment increased the expressions of phosphorylated IκBα and NF-κB, and the ratios of pIκBα/IκBα and pNF-κB/NF-κB increased, indicating the activation of the NF-κB pathway by EHFD. CBD treatment decreased the expressions of pIκBα, pNF-κB, the ratios of pIκBα/IκBα, and pNF-κB/NF-κB, showing the inhibition effect of CBD on NF-κB activation, suggesting the effect of CBD on EHFD-induced inflammation is associated with the activation of the NF-κB pathway.

### CBD Treatment Suppressed NLRP3 Inflammasome Activation and Pyroptosis in EHFD-Fed Mice

**FIGURE 5:**
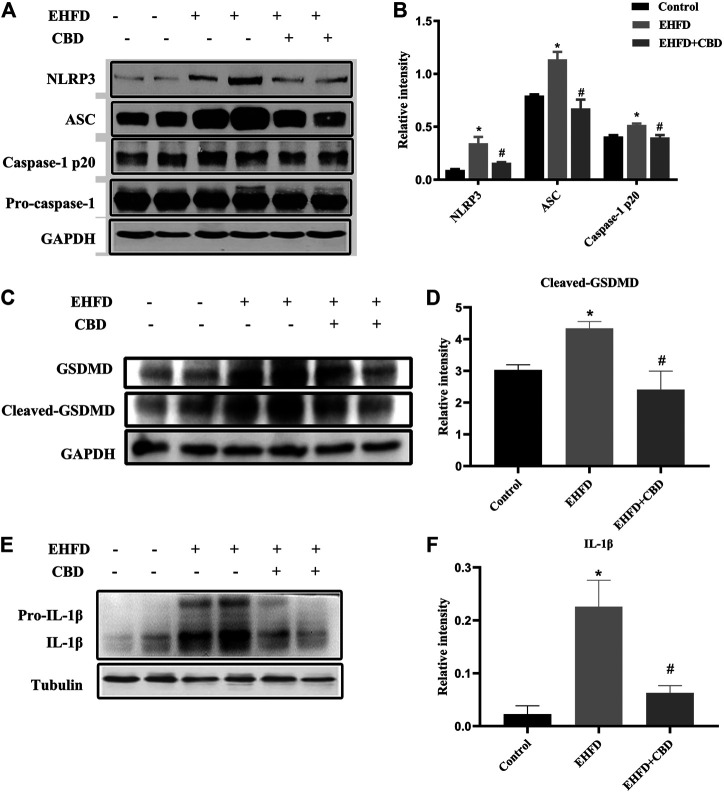
CBD treatment suppressed NLRP3 inflammasome activation and pyroptosis in EHFD-fed mice. Mice were fed ethanol plus high-fat high-cholesterol diet for 8 weeks with or without CBD treatment. Control mice were fed a standard chow diet. Western blot analyses of NLRP3, ASC, pro-caspase-1, and caspase-1 p20 **(A)**. Quantification of expressions of NLRP3, ASC, and caspase-1 p20 **(B)**. Western blot analyses of GSDMD and cleaved GSDMD **(C)**. Quantification of relative expressions of cleaved GSDMD **(D)**. Western blot analyses of IL-1β and pro-IL-1β **(E)**. Quantification of expression of IL-1β (F). *p < 0.05 vs the control group; #*p* < 0.05 vs the EHFD group.

NLRP3 inflammasome activation may lead to GSDMD-driven pyroptosis, which plays a key role in the occurrence and development of liver diseases (Guo et al., 2019). We explored whether CBD could attenuate NLRP3 inflammasome activation and the subsequent pyroptosis in livers. As shown in Figures 5A, B, EHFD diet significantly increased NLRP3, ASC, and caspase-1 p20 protein levels, which indicates NLRP3 inflammasome activation, while their expressions were significantly decreased by CBD treatment.

As certain inflammasomes trigger the inflammatory form of cell death, we then assessed the cleavage of GSDMD, which is the executor of cell pyroptosis. As shown in Figures 5C, D, the expression of cleaved GSDMD in the EHFD group was approximately 1.4 times higher than that in the control group. CBD treatment significantly lowered the protein expression of cleaved GSDMD in livers. Meanwhile, the protein levels of downstream inflammatory cytokine IL-1β were reduced by CBD treatment (Figures 5E, F). Thus, our results suggested a novel mechanism of CBD attenuating liver inflammatory response by inhibiting the NLRP3 inflammasome–pyroptosis pathway.

## Discussion

Metabolism-related liver diseases have been severe public health burdens worldwide. The early stage of liver diseases is reversible through alcohol abstinence, nutritional intervention, or medical treatment (Louvet and Mathurin, 2015). Recently, hepatoprotective property of CBD has been reported in several research studies both in the ALD mice model and cell model (Yang et al., 2014; Wang et al., 2017; Huang et al., 2019). In this study, we employed a liver-damaged mouse model induced by EHFD, which was used to imitate the alcoholics with Western dietary pattern (Gäbele et al., 2011). Our results demonstrated that CBD exerted a beneficial effect in preventing liver injury induced by EHFD diet; 1) CBD reduced lipid accumulation, liver injury, and oxidative stress induced by EHFD diet; 2) CBD attenuated inflammation, by suppressing activation of NF-κB and activation of the NLRP3 inflammasome; and 3) CBD inhibited cleaved GSDMD formation induced by EHFD treatment. Herein, our research is the first to have examined the function of CBD, which protected the liver from injury and inflammation induced by EHFD diet, by regulating the NF-κB–NLRP3 inflammasome–pyroptosis pathway.

Oxidative stress plays a key part in the pathogenesis of liver injuries by alcohol or high-fat diet (Xu et al., 2017). Acute or chronic alcohol drinking or high-fat diet may induce oxidative stress by increasing reactive oxygen species (ROS) production or decreasing antioxidant enzyme activities (Wei et al., 2019), resulting in liver injury. Much evidence indicated that CBD can possess antioxidant properties. We have reported previously that CBD can prevent fatty liver induced by alcohol *via* inhibiting oxidative stress and activating the autophagy pathway (Yang et al., 2014). In line with these reports, we found that CBD lessened EHFD-induced oxidative stress, which was shown by the increasing levels of MDA, the decreasing ratio of GSH/GSSG, and the reduction of the activity of SOD. The increased ratio of GSH/GSSG may contribute to the protection of livers from oxidative stress induced by EHFD. These indicated that part of the hepatoprotective effect of CBD derives from its antioxidative effect.

Chronic alcohol abuse or long-term high-fat diet can lead to augmentation of enteric permeability, hepatocyte injury, and activation of immune cells, resulting in the release of LPS, pro-inflammatory chemokines, and DAMPs (Kawaratani et al., 2017). Consequently, these factors may stimulate the inflammatory response, which is responsible for the progression of liver injuries. Inflammation in the liver has been proven to be a major contributor to the progression of liver disease. Importantly, it has been reported that CBD exerted a noteworthy anti-inflammatory effect in many disorders. Moreover, our laboratory and others previously found that CBD alleviated liver injury of NAFLD by suppressing NF-κB and the NLRP3 inflammasome (Mridha et al., 2017; Huang et al., 2019). In this study, besides inhibiting liver injury, we also found that CBD treatment prominently alleviated the inflammation induced by EHFD shown by counterbalancing the increment of macrophages in the liver induced by EHFD diet. Elevated infiltration of macrophages into the liver is an established pathological alteration of inflammation Zhang et al. (2018), resulting in the release of various cytokines, such as TNF-α, IL-1β, and MCP-1, which could exacerbate liver damage (Xiang et al., 2015). In line with this, we found that CBD treatment actually ameliorated the expression of TNF-α, IL-1β, and MCP-1 in the livers of EHFD-treated mice.

Although these detrimental factors can stimulate inflammatory response through diverse pathways, the most important one of these pathways is characterized by the activation of NF-κB. While these agonists combine with different receptors (Boaru et al., 2015), such as tumor necrosis factor receptor, toll-like receptors (TLRs) (Xiang et al., 2015), IL-1β receptor, and the cytosolic pattern recognition receptor (NOD2), the IκB kinase (IKK) subsequently is catalyzed into an active form, as well as NF-κB (Liu et al., 2017). Unsurprisingly, EHFD feeding caused the activation of IKK and NF-κB. However, CBD visibly inhibited these alterations. Of the NF-κB pathway network, the activation of the NLRP3 inflammasome is a major contributor to chronic liver diseases (Wu et al., 2017). Upgradation of NLRP3 by active NF-κB (signal 1), the so-called priming step, is the first and indispensable process in the activation of the NLRP3 inflammasome (Volt et al., 2016). Followed by the stimulation of signal 2, ACS is recruited and pro-caspase-1 self-catalyzed into caspase-1 (Volt et al., 2016), the mature form. Subsequently, caspase-1 cleaved pro-IL-1β into mature IL-1β and GSDMD into cleaved GSDMD, resulting in inflammation and injury (Fabio et al., 2002; TingTing et al., 2010). In accordance with the activation of NF-κB, the NLRP3 inflammasome and GSDMD were also activated by EHFD diet, CBD treatment expectedly downgraded the level of NLRP3 and ASC and reduced the activation of pro-caspase-1 and mature IL-1β.

In this study, we employed a liver damage model induced by ethanol combined with high-fat diet, simulating the social drinking action and impactful Western diet pattern. In this study, we only examined the effect and explored the mechanism in the mice model but did not confirm the molecular mechanism in hepatocytes. Further cell experiments with certain gene knockdown in the NF-κB–NLRP3 inflammasome–pyroptosis pathway are warranted to confirm the molecular mechanisms.

## Conclusion

The current study indicates that CBD protects the liver against EHFD-induced liver inflammatory reactions, potentially *via* inhibiting NLRP3 inflammasome activation and pyroptosis. Future investigation toward elucidating the mechanisms underlying pyroptosis will benefit our understanding of the beneficial effects of CBD.

## Data Availability

The original contributions presented in the study are included in the article/supplementary material; further inquiries can be directed to the corresponding authors.
